# PRIME-IPD SERIES Part 1. The PRIME-IPD tool promoted verification and standardization of study datasets retrieved for IPD meta-analysis

**DOI:** 10.1016/j.jclinepi.2021.05.007

**Published:** 2021-08

**Authors:** Omar Dewidar, Alison Riddle, Elizabeth Ghogomu, Alomgir Hossain, Paul Arora, Zulfiqar A Bhutta, Robert E Black, Simon Cousens, Michelle F Gaffey, Christine Mathew, Jessica Trawin, Peter Tugwell, Vivian Welch, George A Wells

**Affiliations:** a Bruyère Research Institute, University of Ottawa, 85 Primrose Ave, Ottawa, Ontario, K1R 6M1, Canada; b School of Epidemiology and Public Health, University of Ottawa, 600 Peter Morand Crescent, Ottawa, Ontario, K1G 5Z3, Canada; c Department of Medicine (Cardiology), The University of Ottawa Heart Institute and University of Ottawa, 40 Ruskin Street, Ottawa, Ontario, K1Y 4W7, Canada; d Dalla Lana School of Public Health, University of Toronto, 155 College St Room 500, Toronto, Ontario M5T 3M7, Canada; e Centre for Global Child Health, Hospital for Sick Children, 555 University Ave, Toronto, Ontario, M5G 1X8, Canada; f Institute for Global Health & Development, Aga Khan University, South-Central Asia, East Africa & United Kingdom, Karachi, Pakistan; g Department of International Health, Johns Hopkins Bloomberg School of Public Health, 615N Wolfe St Suite E8545, Baltimore, MD, 21205, USA; h Department of Infectious Disease Epidemiology, London School of Hygiene and Tropical Medicine (LSHTM), Keppel Street, London, WC1E 7HT, UK; i Clinical Epidemiology Program, Ottawa Hospital Research Institute, 501 Smyth Rd, Ottawa, Ontario K1H 8L6, Canada; j Department of Medicine, University of Ottawa Faculty of Medicine, Roger Guindon Hall, 451 Smyth Rd #2044, Ottawa, Ontario, K1H 8M5, Canada; k WHO Collaborating Centre for Knowledge Translation and Health Technology Assessment in Health Equity, Bruyère Research Institute, 85 Primrose Ave, Ottawa, Ontario, K1R 6M1, Canada; l Cardiovascular Research Methods Centre, University of Ottawa Heart Institute, 40 Ruskin St, Ottawa, Ontario, K1Y 4W7, Canada

**Keywords:** Data replication, Meta-analysis, Individual participant data, Imputation, Research methodology, Data Preparation, Evidence Synthesis, Randomized Trials

## Abstract

**Objectives:**

We describe a systematic approach to preparing data in the conduct of Individual Participant Data (IPD) analysis.

**Study design and setting:**

A guidance paper proposing methods for preparing individual participant data for meta-analysis from multiple study sources, developed by consultation of relevant guidance and experts in IPD. We present an example of how these steps were applied in checking data for our own IPD meta analysis (IPD-MA).

**Results:**

We propose five steps of Processing, Replication, Imputation, Merging, and Evaluation to prepare individual participant data for meta-analysis (PRIME-IPD). Using our own IPD-MA as an exemplar, we found that this approach identified missing variables and potential inconsistencies in the data, facilitated the standardization of indicators across studies, confirmed that the correct data were received from investigators, and resulted in a single, verified dataset for IPD-MA.

**Conclusion:**

The PRIME-IPD approach can assist researchers to systematically prepare, manage and conduct important quality checks on IPD from multiple studies for meta-analyses. Further testing of this framework in IPD-MA would be useful to refine these steps.

**What is new?**

**Key findings**
• The multi-step approach that can be used to manage IPD for analysis from multiple studies involves the following stages:
• Processing
• Replication
• Imputation
• Merging
• Evaluation

**What this adds to what is known?**
• PRIME-IPD provides a formalized step-by-step approach to verify and prepare individual participant data from multiple studies for meta-analysis, thus adding to available guidance on evidence synthesis.

**What is the implication and what should change now?**
• The synthesis of IPD from multiple trials provides a powerful approach to control for confounding and investigate effect modification at the individual level. However, a principled and systematic way to build the analytic dataset with requisite checks for data quality, is needed to ensure these benefits are realized.
• Further testing of this framework to assess feasibility and applicability to other reviews may refine this model.

## 1 Introduction

Clinical decision-makers increasingly rely on systematic reviews and meta-analyses because they collate, critically appraise and synthesize all relevant evidence on a particular question [1]. Individual participant data meta-analysis (IPD-MA) is considered the gold standard in systematic reviews since it enables effect modification analyses using individual-level data [2]. IPD-MA is carried out by collecting raw individual participant data from all eligible studies for which the data are available. The data are then pooled and reanalyzed simultaneously [2,3]. IPD-MA has advantages over conventional aggregate data meta-analysis (AD-MA), such as minimizing selective reporting bias and allowing better characterization of subgroups and outcomes as well as data quality assessment [4], [5], [6].

IPD are usually acquired by directly contacting the study authors [7]. However, there are multiple barriers to the smooth retrieval of datasets [8,9]. Authors may be hesitant about sharing their datasets due to concerns about how the data will be used, data security and other issues. In the process of including IPD, studies may be subjected to reanalysis. Data sharing hesitancy may stem from apprehensions around having their data scrutinized and re-analyzed [7]. This highlights the importance of developing standardized measures for assessing data quality.

Furthermore, IPD datasets sponsored by industry, such as pharmaceutical and medical device companies, are rarely available and accessible [10]. A systematic review exploring the retrieval of IPD for IPD-MA shows that over 20 years, only 25% of systematic reviews were able to obtain all of the relevant datasets [11]. Over half of the reasons for the unavailability of IPD was the loss of datasets, which highlights the need for improvements in data collection and archiving. Polanin et al. [7] have suggested that using a data-sharing agreement document may alleviate concerns related to data sharing, increasing the likelihood of data sharing and promoting transparent, academic collaboration.

Managing and preparing IPD is resource intensive and time consuming [3,[12], [13], [14]]. IPD datasets differ in their naming conventions, data structure and file formats. Older datasets require even more maintenance as they tend to not be recorded to the current standards. Tudur Smith et al. [15] reported the multiple challenges in data preparation, such as the absence of a summary of variables, data collection in separate files and software incompatibility, resulting in the consumption of extensive amounts of time and resources. Despite the increasing interest in performing IPD-MA and initiatives to improve methods through the Cochrane handbook as well as guidance provided by credible IPD working groups [16], [17], [18], [19], there is an absence of a comprehensive and formal approach to collect, verify and analyze the individual level data.

This paper aims to describe the approach we developed and illustrates its value when applied, on an IPD-NMA for mass deworming for children [20,21].

## 2 Methods

A project advisory group composed of experts in IPD, statisticians, methodologists and systematic reviewers was established to develop a systematic approach to collate and prepare individual participant data for analysis. Prior to developing this approach, we reviewed relevant guidance from the Cochrane Handbook [16], Get Real IPD Working Group, Cochrane Multiple Interventions Group [18] including their library and the Cochrane Methods IPD Meta-Analysis Group [19]. We itemized and categorized components of the relevant guidance and reached consensus on the development of a 5-step approach to prepare the IPD data post-acquisition from study authors. We illustrated the application of this approach to an IPD-NMA on deworming [20,21].

## 3 Results

Based on the literature and consensus process we developed a five stage systematic approach for the preparation and the conduct of an IPD-NMA systematic review. They are:

1. *Processing* of the datasets
2. *Replication* of published data tables
3. *Imputation* of missing data
4. *Merging* datasets
5. *Evaluation* of data heterogeneity

Table 1 provides an overview of the steps undertaken at each stage. The overview is followed by an illustrative example of the methodology using our IPD-NMA of mass deworming interventions for children in low-resource settings [20,21].

**Table 1:** Checklist items for PRIME-IPD tool

PRIME:	Items
Processing	• Convert data into a single format for statistical program of choice • Compare the total number of participants in the acquired datasets to those reported in published studies • Verify the presence of the variables of interest in the acquired dataset • Standardize variable names across datasets • Identify and standardize the measurement scales used to report the variables of interest • Identify and standardize coding for missing values • Identify and correct any implausible values that may result from data conversion
Replication	• Recalculate reported descriptive and summary statistics using the acquired datasets • Calculate the standardized difference to quantitatively assess the difference between the replicated and published results • If the standardized difference is > 10%, investigate and address potential causes
Imputation	• Assess the appropriateness of conducting imputation of missing data using missing data theory • If multiple imputation is conducted, carefully consider the number of imputations to be run
Merging	• Ensure in processing step that variable order and codes are correct • Merge the imputed datasets into a single, pooled dataset, taking into consideration the number of imputed datasets, if appropriate
Evaluation	• Assess continuous variables for normality by residual analysis either visually or by statistical tests • If required, calculate new variables for standardized comparison of effects

### 3.1 Processing of datasets

The first stage of data processing is to standardize the format of the datasets that will be included in the final IPD analysis. However, several challenges may arise. Acquired datasets may be in different formats (e.g., SAS vs. SPSS), different variable names may be used for the same measure, different scales may be used to report the same measure (e.g., hemoglobin may be reported in grams per liter or grams per decilitre), and some indicators or values may be missing for some individual studies. Missing data may be indicated by different symbols or notations (such as “−99″). Data dictionaries may not be available for all datasets. Consequently, we recommend the following steps in the ‘Processing’ stage to overcome these challenges:

1. Convert each acquired dataset to a preferred standardized format (e.g., SAS, STATA). The format should be chosen based on facilitating easy data manipulation. This format may or may not be the format used for the eventual analyses.
2. Compare the total number of observations in the received datasets to those reported in the published studies (or global trials registers if publications are not available). In the event of a mismatch, determine the cause of the discrepancies. In event of mismatch, contact the authors to understand reason for discrepancy.
3. Verify that the variables of interest are available in the acquired datasets by referring to accompanying data dictionaries. In their absence, contact the primary authors of the studies for the information required.
4. Create a master list of individual dataset variable names mapped to the variable name of choice. Rename all variables of interest across the datasets to have common variable names.
5. For continuous variables, identify the variables’ scales of measurement and identify any datasets that may need to have values converted to the preferred standard using appropriate conversion formula(e). Determine whether the categories of the categorical variables need to be regrouped or separated into dummy variables.
6. Identify any missing values in the datasets and how they are identified in the dataset (e.g., blank cells, symbols). Confirm that the blank cells are missing values and not due to a conversion error by comparing the percentage of missing values per variable in the acquired and converted datasets and standardize across datasets. Similar considerations may exist for data considered not applicable.

### 3.2 Replication of published data tables

The second step is to replicate the data tables reported in the published studies. Since reproducibility is an anchor in scientific research [22], [23], [24], it is essential to check that the processed datasets are consistent with the analyzed datasets in the published papers. This step will provide an additional check on data quality and fidelity to the acquired study and increase confidence that the datasets were processed correctly. Discrepancies between the replicated and published results are often expected [25,26]. Challenges in the replication process include discrepancies in the number of participants or units of analysis between the published paper and the acquired datasets and lack of reporting on statistical methods. The following steps are proposed to assist in minimizing these challenges:

1. Calculate and compare the descriptive statistics from the processed datasets to the published results. For example, the percentage of females enrolled, age of participants, and pre-existing health conditions.
2. Calculate and compare baseline and endline summary statistics for the outcomes of interest from the processed datasets to the published results using the same analytic methods reported in the published article.
3. Calculate the standardized difference between the descriptive and summary statistics of the published studies and the replicated results. We referred to the absolute standardized difference criterion of 10% proposed to assess baseline imbalance to assess the magnitude of difference between replicated and published results. We chose the criterion of 10% as an indicator of discrepancy between published and replicated results based on previously proposed thresholds [27], [28], [29], [30]. The standardized difference can be calculated as follows:
  $$\frac{\left(difference\;between\;replicated\;and\;published\;results\right)}{\sqrt{Variance\;\left(difference\;between\;replicated\;and\;published\;results\right)}}$$

Note: Variance is calculated assuming independence of replicated and published results.

### 3.3 Imputation of missing data

Missing data are inevitable in clinical research [31]. Complete case analysis, ignoring participants with missing data, has the potential to bias results [32] and can reduce a study's precision and power due to a smaller sample size. Imputation may be considered to redress missing data, depending on the amount and type of missingness in the processed datasets and according to missing data theory [33]. If multiple imputation is implemented, carefully consider the number of imputations to be run [34], taking into consideration that a greater number of imputations will result in longer computing time [32].

### 3.4 Merging datasets

The merging of datasets in this context refers to the vertical merging of rows (or observations) of two or more datasets. All datasets to be combined in the merge step should already have the same variables (or columns) following the processing step. Different statistical programs will have different names for this command, or multiple ways that datasets may be merged. For example, in SAS you may use concatenation in the DATA step command, or the APPEND procedure (SAS Institute Inc., Cary, NC, USA). Readers should follow the guidance for merging provided by their statistical software program, as specific steps can vary. It is important to ensure that you can identify from which original study each observation belongs after the merge step. This can be done by creating a variable for study name. Alternatively, in Stata, you may employ the “generate” option [35] to create a variable identifying from which dataset each observation originally came. Observations from imputed datasets will also need to be correctly labelled according to their original study and imputation number.

### 3.5 Evaluation of data heterogeneity

Prior to the conduct of a pooled analysis, an assessment of the merged dataset's heterogeneity and distribution may be explored to inform statistical methods and interpretation of results. Further, authors may need to calculate new variables for the standardized comparison of effects. We suggest the following:

1. Test data distribution by residual analysis for continuous variables either visually, by preparing bar charts, or by parametric statistical tests [36]. Comparisons can be implemented between study arms to appraise the randomization of participants in each group and identify differences between study groups.
2. Create new variables needed for analysis (e.g., “dummy variables” for categorical variables). This step is needed if there are any variables which need to be calculated based on existing variables in the merged dataset (e.g., body mass index may be calculated using existing data on the height, weight, age and sex of participants).

## 4 PRIME application

We report our experience using PRIME-IPD for preparing data for an individual participant data network meta-analysis of mass deworming interventions for children in low-resource settings [20,21] as an exemplar in Table 2. The appended table shows the value-added of each step in verifying and standardizing the acquired data for use in an IPD-NMA.

**Table 2:** Application of PRIME-IPD in the context of a deworming systematic review

PRIME:	Problem	Application
Processing	Incomplete and missing data dictionaries	We used a list of analysis variables to request data since it identified which variables we needed and reviewed the dataset files along with the data dictionaries. Correspondence with authors was helpful in preparing datasets lacking dictionaries.
	Identification of missing variables of interest	We documented the choice of outcome measures for studies that collected data at multiple time points and identified four out of 11 studies which did not report the primary outcomes of interest in their published manuscripts, but they did collect this data and provided it in their dataset.
	Use of different measurement methods	We evaluated the measurements for helminth egg counts were provided. We identified various measurement methods used between authors in terms of the number of egg samples taken and how they were collected. We selected the most common method and standardized it across all included studies.
	Identification of conversion errors	We identified the presence of implausible values that required conversion before analysis such as zeros coded 0.99 and 9999.
Replication	Inexact number of participants in the datasets compared to reported	The authors provided full datasets, including children who were excluded from the analysis due to missing baseline measures (e.g., missing stool samples). Replication allowed us to verify that these children were excluded from the analyses in the published papers.
	Incorrect treatment labels	By means of replication, we found that the labels in the dataset from authors did not match the labels in the published paper. Correspondence with the authors allowed us to correct these labels and replicate the analyses
	Uncorrected variables in the provided datasets	Hemoglobin concentration need to be corrected if measured in individuals living in areas 1000 m above sea level, since lower oxygen levels, result in higher hemoglobin concentrations in the blood. Hemoglobin was not adjusted for in two studies’ datasets which were carried out in areas 1000 m above sea level, so the Hemoglobin concentration values obtained when replicating were larger than the reported [37].
Imputation	Studies with missing data	For each study included in the IPD analysis, we calculated the percentage of missing data for each variable of interest. Consequently, we assessed the distribution of the missing variables to assess if imputation was appropriate. We imputed the eligible studies that had less than 50% of missing data and assumed data were missing at random, creating five imputed datasets per study. We used complete case analysis for studies with more than 50% of missing data as part of sensitivity analyses only.
Merging	Correctly combining multiple datasets	A separate variable was created to identify each observation's original study and imputation number (ranging from one to five). We sorted datasets by that identifier and used MERGE used the command in SAS (9.4) to combine the imputed datasets into a new dataset.
Evaluation	New variable calculation	Growth standards have varied over the years. We used WHO anthropometric software to calculate BMI for age, weight for age and other growth standards in relatively older studies to combine with the other studies. The Anthropometric calculator in the software also operates similar SAS by tagging implausible weight and height values.

## 5 Discussion

This paper details a methodology for the preparation of data for IPD-MA composed of five steps: Processing, Replication, Imputation, Merging and Evaluation. Standardization of included datasets is performed in the processing step, followed by verification of datasets through data replication. To deal with missing data, we propose imputation if appropriate, according to missing data theory. Following the merging of the processed datasets and prior to conducting analyses using the merged dataset, we suggest assessing heterogeneity across the variables in the evaluation step and creating any new variables that are required for analysis. Many aspects within PRIME-IPD will help formulate the Statistical Analysis Plan (SAP) for IPD. Subsequent to data preparation, synthesizing study data is needed to assess the intervention effects. This step bears its own series of barriers and challenges with guidance provided elsewhere [6,13,17,38].

The five step approach of PRIME-IPD is a comprehensive composite of previous research methods and guidance for IPD. The Cochrane Handbook version 5 [16] highlights the importance of recoding variables during data preparation but does not detail procedures to prepare the dataset for IPD analysis. The “get-real” review conducted by Debray et al. provides insight on how to distinguish between different missing data scenarios but does not provide suggestions when considered, may improve the robustness of the imputation process [17].

An important aspect of our approach is in including a data replication step, which can help verify what the authors report in their published studies and identify any errors present in the processed datasets. Replicating the acquired studies’ descriptive and summary statistics helps to identify critical assumptions made by the original investigators and data inconsistencies in the acquired datasets. This process adds to the robustness of the IPD analysis conducted by the investigators. There are additional proposed benefits to re-analyzing the datasets as conducted by the original investigators such as ensuring complete, accurate and unbiased reporting of results [39]. However, the additional time and cost that may be incurred to conduct the replication should be considered [40]. The replication process can become unwieldy with a large number of studies. We also acknowledge that the PRIME-IPD methodology is a lengthy process. However, based on firsthand experience, we found the benefits outweigh the costs because we were able to identify and correct data problems before pooling and data synthesis.

The success of conducting IPD-MA does not solely depend on the preparation of datasets for analysis, but heavily depends on the ability to retrieve datasets from authors. There are several challenges to accessing IPD, as it is often unavailable even upon request from authors [41], [42], [43]. Less than 50% of IPD-MA systematic reviews published between 1987 and 2015 succeeded in retrieving at least 80% of their selected studies [7]. Therefore, there is a crucial need to build confidence and trust among investigators using data sharing agreements, which have been shown to increase the likelihood of a response [7,44] and using investigator collaboratives [7]. Improving IPD access coincides with initiatives to make data available through online data repositories (public or private) such as Vivli [45] and OpenTrials [46, [47], [48], [49], [50], [51]]. The role of data repositories in facilitating IPD analysis remains limited in terms of the curation of datasets for analysis. Investigators are requested to upload dictionaries, but they are usually incomplete, and datasets lack organization with major heterogeneity between studies [52]. The PRIME-IPD approach overcomes these hurdles when dealing with several datasets through providing a systematic approach to preparing data for analysis, including verification of terms with authors if needed. Data-sharing repository services may address this limitation in the future by unifying policies and systems.

## 6 Conclusion

PRIME-IPD proposes a systematic approach to the preparation and verification of individual participant datasets. Combining PRIME-IPD with best practices in acquiring datasets from authors, such as the use of data-sharing agreements, and offering appropriate acknowledgement and incentives, may improve efficiency in conducting IPD analysis. Nonetheless, the PRIME-IPD approach requires further testing in different settings and may be require adaptations in specific scenarios.

## Appendix Supplementary materials

Image, application 1
