# Supplementary material for: PRIME-IPD SERIES Part 1. The PRIME-IPD tool promoted verification and standardization of study datasets retrieved for IPD meta-analysis
Source: J Clin Epidemiol. 2021 Aug;136:227–34. doi: 10.1016/j.jclinepi.2021.05.007 (PMC8442853; doi:10.1016/j.jclinepi.2021.05.007)
Supplement: Supplementary file 1 [file mmc1.docx]

**Appendix 1. Example of a data replication table.**

|  | Control | | | | | Intervention | | | | |
| --- | --- | --- | --- | --- | --- | --- | --- | --- | --- | --- |
|  | Published | | **Replication** | |  | Published | | **Replication** | |  |
|  | **(N = 1095)** | | **(N = 1095)** | |  | **(N = 1084)** | | **(N = 1084)** | |  |
| **Individual Characteristics** |  | St. Dv. |  | St. Dv. | Standard Diff |  | St. Dv. |  | St. Dv. | Standard Diff |
| Age | 10.61 | 0.844 | 10.61 | 0.87 | 0.00 | 10.56 | 0.84 | 10.56 | 0.86 | 0.00 |
| Female (%) | 43.38 | 49.81 | 43.49 | 49.58 | 0.00 | 49.00 | 0.54 | 49.00 | 0.50 | 0.00 |
| **Infection Prevalence** |  |  |  |  |  |  |  |  |  |  |
| Any STH infection (%) | 41.10 | 48.96 | 41.1 | 49.22 | 0.00 | 42.6 | 49.55 | 42.6 | 49.48 | 0.00 |
| *Ascaris* infection (%) | 30.50 | 45.58 | 30.5 | 46.06 | 0.00 | 31.10 | 46.19 | 31.10 | 46.31 | 0.00 |
| *Trichuris* infection (%) | 23.29 | 42.21 | 23.29 | 42.29 | 0.00 | 24.40 | 42.84 | 24.35 | 42.94 | 0.00 |
| Hookworm infection (%) | 1.00 | 10.13 | 1.00 | 9.98 | 0.00 | 0.70 | 8.40 | 0.74 | 8.56 | 0.00 |
| *Ascaris* and *Trichuris* co-infection (%) | 12.97 | 33.77 | 12.97 | 33.61 | 0.00 | 12.9 | 33.60 | 12.92 | 33.55 | 0.00 |
| **Infection Intensity (among samples with positive infection)** |  |  |  |  |  |  |  |  |  |  |
| *Ascaris* infection (epg) | 728.32 | 3400.25 | 728.00 | 3374.92 | 0.00 | 1065.00 | 5440.04 | 1065.00 | 5397.21 | 0.00 |
| *Trichuris* infection (epg) | 55.90 | 297.14 | 55.90 | 295.45 | 0.00 | 71.80 | 399.79 | 71.8 | 396.43 | 0.00 |
